# Enhancement of kefir functionality by adding black elderberry and evaluation of its quality during storage

**DOI:** 10.1002/fsn3.4481

**Published:** 2024-10-04

**Authors:** Ümran Barazi, Seher Arslan

**Affiliations:** ^1^ Department of Food Engineering, Engineering Faculty Pamukkale University Denizli Turkey

**Keywords:** anthocyanin, antioxidant activity, dried elderberry powder, elderberry mash, kefir, physical properties

## Abstract

The research explored the influences of 2% and 4% heat‐treated fresh elderberry fruit mash supplement and 0.5% and 1% dried elderberry powder supplement before and after fermentation in kefir production on certain features of kefir beverage. Antioxidant activity, total monomeric anthocyanin, and total phenolic content were 16.69–75.67 (μmol TE/kg), 14.64–88.20 (mg cyn‐3‐gly/kg), and 167.94–531.33 mg GAE/kg in the samples during storage, respectively. Total phenolic content and antioxidant activity were higher in kefir samples produced with elderberry powder when compared to the other samples. It was determined that L* and b* increased with the decrease in the fruit supplement in kefir production, and L* and b* values were mostly higher in kefirs with fruit supplement before fermentation. The highest ACE inhibitor activity was observed in the kefir sample (1‐DEPre sample) with 1% dried elderberry supplement before fermentation on the first day of storage. It was determined that the general appreciation scores were above 3, and the samples produced mostly with dried elderberry powder were appreciated more when compared to the samples produced with fresh elderberry.

## Graphical abstract

This study aimed to investigate the quality characteristics of kefir supplemented with fresh elderberry fruit mash and elderberry powder as a source of natural color and due to its rich nutritional content. Key findings show a positive association of some sensory properties, antioxidant activity, and total phenolic content with samples containing elderberry powder. The sample with 4% postfermentation elderberry mash received the highest total monomeric anthocyanin content at the beginning of storage. Elderberry powder could be used in kefir production successfully.

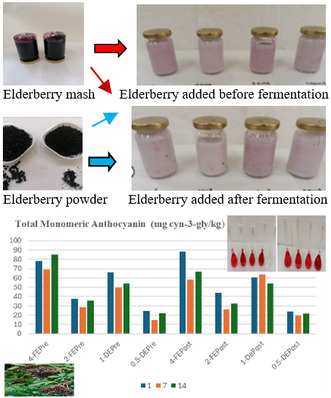

## 1 INTRODUCTION

Kefir is a low‐alcohol, acidic probiotic milk drink manufactured by fermenting milk at ambient temperature (Saleem et al., 2023). Since kefir production entails homolactic, heterolactic, and alcoholic fermentation, the primary end products include lactic acid (0.8%), ethanol (1.0%), and carbon dioxide. The taste of kefir depends on diacetyl, acetaldehyde, and organic acids. Specifically, the characteristic taste of kefir is mainly obtained with the optimal 3:1 diacetyl/acetaldehyde ratio (Gentry et al., 2023).

Kefir is a fermented milk product produced with kefir grains or cultures (Basaran & Telci, 2022). The size of the kefir grains varies between 1 and 4 cm, their color varies between white and light yellow, and their shape resembles small cauliflowers. This gelatinous and slimy structure includes a natural exopolysaccharide matrix (EPS), kefiran, and proteins, where lactic acid bacteria (LAB), yeasts, and acetic acid bacteria (AAB) coexist symbiotically (Azizi et al., 2021). The microflora in kefir varies based on certain factors such as the composition of the kefir grains or culture, culture medium, fermentation temperature, fermentation duration, milk composition, milk type, and storage conditions. All of these influence its structure. Kefir microbiota include LAB, AAB, and yeasts (Gocer & Koptagel, 2023).

Kefir includes vitamins and essential amino acids that help healing of the body and maintenance of its functions, and sufficient easily digestible proteins. Kefir consumption has various advantages. Kefir has been used for its therapeutic properties in several diseases and disorders (Saleem et al., 2023). Various studies investigated the antimicrobial, anticarcinogenic, antioxidant, lactose intolerance mitigating, cholesterol regulating, antihypertensive, and wound healing properties of kefir (Chen et al., 2007; Hertzler & Clancy, 2003; Huang et al., 2013; Huseini et al., 2012; Kesenkaş et al., 2017; Monteiro et al., 2020; Yilmaz‐Ersan et al., 2018). Although kefir is a probiotic drink with high nutritional value, it does not appeal to consumers of all ages due to its acidic taste. Thus, recent studies aimed to enrich kefir with various fruit, flavor, and vegetable juice supplements (Corona et al., 2016; Kabakcı et al., 2020; Najgebauer‐Lejko et al., 2021).

*Sambucus nigra* L. is a small, 4–12 m tall deciduous tree or shrub that grows in natural habitats in Asia, North America, Europe, North Africa, New Zealand, and Australia. *S. nigra* L., also called European wild black elderberry, elderberry, European elderberry, and European black elderberry, is a member of the Adoxaceae family, which was previously a member of Caprifoliaceae (Stępień et al., 2023). The fruits of the species are spherical, small (0.4–0.6 cm in diameter), and grow in large 15 to 22 cm clusters (Pascariu & Israel‐Roming, 2022). The elderberry fruit is a spherical drupe that contains 3–5 pressed seeds. Elderberry fruits are initially green, but the color changes from red to black/purple during ripening, their skin is shiny, and they contain plenty of juice. *S. nigra* L. blooms in May and June, while fruits generally ripen between July/August and October (Stępień et al., 2023).

Elderberry is nutritional and rich in vitamins C, A, iron, and potassium minerals, and a good antioxidant source. Elderberry also has high bio‐pharmacological content, including flavonoids, phenolic acids, anthocyanins, terpenes, and alkaloids (Terzić et al., 2022). The ripeness of the fruit affects anthocyanin content (ripe fruits have higher content), especially cyanidin‐3‐sambubioside. This affects the cyanidin‐3‐sambubioside−5‐glucoside, cyanidin‐3‐sambubioside, and cyanidin‐3‐glucoside content (Pascariu & Israel‐Roming, 2022). Elderberry has antioxidant, anti‐inflammatory, antimicrobial, antidiabetic, and neuroprotective properties. Elderberry products have special immune properties and are preferred in the relief of cold and flu symptoms (Pascariu & Israel‐Roming, 2022; Terzić et al., 2022).

Elderberry flowers and berries are employed in the food industry as an alternative material in pies, punches, jellies, jams, ice creams, yogurts, wines, herbal teas, liqueurs, juices, and as flavoring (Senica et al., 2016; Viapiana & Wesolowski, 2017). Various studies employed elderberry in meat products, gluten‐free wafers, pastas, croissants, and as a food color (Cordeiro et al., 2020; Pascariu & Israel‐Roming, 2022; Rozylo et al., 2019; Sun‐Waterhouse, Jin, & Waterhouse, 2013). Elderberry fruits have been used internationally in medicine and as a food supplement (Veberic et al., 2009).

Supplementing kefir with elderberry mash and powder for nutritional and functional benefits could be an effective strategy to improve consumer appreciation and health. The present research purposed to state the effect of 2% and 4% fresh elderberry fruit mash and 0.5% and 1% dried elderberry powder supplementation before and after fermentation on the physical, composition, biological activity, microbiological, rheological, and sensory properties of kefir during 14‐day storage.

## 2 MATERIALS AND METHODS

### 2.1 Materials

In kefir production, UHT cow milk was procured from a local market. Dried and fresh elderberries were procured from the Ministry of Agriculture and Forestry, Atatürk Central Horticulture Research Institute (Yalova, Turkey). Elderberry powder was obtained with the separation of the dried elderberry fruit stems and grounding the product. Elderberry mash was obtained by separating the stems and rotten fruits from the fresh elderberry fruit and crushing the fruits. The crushed elderberry fruit was heated up to 90°C and kept at that temperature for 1–2 min. Lyophilized kefir culture was employed for fermentation (MYStarter KF 1 freeze‐dried kefir culture DIC‐direct inoculation culture, *Lactobacillus lactis* spp. *lactis* biovar *diacetylactis*, *Lactococcus lactis* spp. *lactis*, *Lactobacillus brevis, Leuconostoc* spp., and *Saccharomyces cerevisiae*) and procured from Maysa (Istanbul, Turkey).

### 2.2 Kefir production

Fresh elderberry stems were separated, and the fruits were washed. Two hundred milliliters of drinking water were added to every 1 kg of chopped fruit in a blender, and the product was heated to 90°C for 1–2 min. The hot mash product was transferred into a glass jar and stored at +4°C for a day. Dried elderberry fruit was powdered. The milk was heated up to 25°C and dried elderberry fruit powder and elderberry mash, as well as the kefir culture, were added at this temperature to obtain the kefir samples with fruit supplementation. After the fruit and culture were added, the milk was incubated at 23°C for approximately 22 h until the pH was 4.4. After incubation, the coagulated products were mixed, and kefir samples were transferred to glass jars.

For postfermentation fruit‐supplemented kefir samples, kefir culture was added to the milk at 25°C, and the milk was incubated at 23°C for approximately 22 h until the pH was 4.4. After the incubation, the plain kefir was homogenized by adding the predetermined rates of mash or dried elderberry powder. Fruit kefirs were transferred into glass jars and stored at +4°C. The kefir supplementation formulations with various forms of elderberry fruit are presented in Table 1.

**TABLE 1:** Formulations of kefir samples produced with various forms of elderberry fruit.

Sample	Property
Control	Plain kefir
4‐FEPre	Prefermentation 4% fresh elderberry supplementation
2‐FEPre	Prefermentation 2% fresh elderberry supplementation
1‐DEPre	Prefermentation 1% dried elderberry supplementation
0.5‐DEPre	Prefermentation 0.5% dried elderberry supplementation
4‐FEPost	Postfermentation 4% fresh elderberry supplementation
2‐FEPost	Postfermentation 2% fresh elderberry supplementation
1‐DEPost	Postfermentation 1% dried elderberry supplementation
0.5‐DEPost	Postfermentation 0.5% dried elderberry supplementation

### 2.3 Chemical analysis

Dry matter in kefir samples and milk was determined with gravimetric, protein Kjeldahl, and fat Gerber methods (Metin & Öztürk, 2002). Titratable acidity was determined with the alkaline titration method (Anonymous, 2002). Sample pH values were evaluated with a pH meter (Hanna HI 2211, USA). The fresh elderberry fruit juice was extracted by crushing the fruit, and the water‐soluble dry matter content was analyzed with a table‐style Abbe refractometer (Bulut et al., 2020). The total dry matter analysis of dried and fresh elderberry fruits was conducted by bringing the 3 g fruit samples in drying containers to a constant weight in a 60 ± 2°C oven with the gravimetric method (Gerçekcioğlu & Yılmaz, 2022).

### 2.4 Evaluation of biological activity

#### 2.4.1 Extraction

Elderberries were extracted with the modified Ferreira et al. (2020) and Silva, Ferreira, and Nunes (2017) methods, and kefir samples were extracted with the modified Kabakcı et al. (2020) method. The analysis of the kefir samples was conducted with 0.25 g dried elderberry fruit powder, 1 g fresh elderberry fruit shredded in a blender, and 10 g kefir sample in falcon tubes. Five milliliters of acidified methanol (with 1% HCl) were added to the elderberry fruits, and 10 mL was added to the kefir samples, and the tubes were mixed in an orbital shaker (WiseShake SHO‐1D) at 200 rpm for 20 min. Falcon tubes were centrifuged in a centrifuge device (Nüve NF 1200 R; Ankara, Turkey) at 10,000 rpm for 5 min. The supernatant collected in the Falcon tube was transferred to a 50‐mL volumetric flask with a Pasteur pipette. Acidified methanol was added to the fruit remaining in the Falcon tube, and the same process was repeated 8 times for fruit samples and 3 times for kefir samples (until the extract became colorless). After all the supernatants were collected, acidified methanol was added to the extract until it reached 50 mL in volume.

#### 2.4.2 Total monometric anthocyanin

The monomeric anthocyanin content was determined with the common pH differential method (Silva, Ferreira, & Nunes, 2017; Yeler, 2021). The extracts were diluted with buffer solution (0.025 M potassium chloride) adjusted to pH 1.0, and the wavelength of the maximum absorbance was determined between 0.4 and 0.6. The wavelength with the maximum absorbance was 520 nm. The extracts were diluted with pH 1.0 and pH 4.5 buffer solutions, and the absorbance was measured spectrophotometrically at 520 nm and 700 nm.

The total monomeric anthocyanin content was calculated with the following formula:

34𝑇M34𝑇𝐴=(34𝐴×34𝑀34𝑀𝑊×D34𝐹×1000)/(34𝜀×34𝑙).

TMA: Total monomeric anthocyanin content (mg/kg or mg/L). A: Absorbance of the extracts. A = (𝐴520−𝐴700) _𝑝𝐻 1,0_ − (𝐴520−𝐴700) _𝑝𝐻 4,5_. MW: Molecular weight of cyanidin‐3‐glucoside (449.2 g/mol). ε: Molar Absorbance (26,900 kg/mol.cm or L/mol.cm). l: Distance (1 cm). DF: Dilution factor.

#### 2.4.3 Total phenolic content

The total phenolic content was determined with the modified Ertan et al. (2017) method and the Folin Ciocalteau (F‐C) method. Before the analysis, Folin Ciocalteau reagent was diluted with pure water at a 1:10 (v/v) ratio, and sodium carbonate was dissolved with pure water at a 75 g/L ratio. One milliliter kefir or elderberry extract samples were transferred into the test tubes, 5 mL Folin Ciocalteau solution was added, and 4 mL sodium carbonate solution was added. After the tubes were stored in the dark at ambient temperature for 2 h, they were observed with a spectrophotometer (Nüve, NF 1200R) at 760 nm, and the findings are presented as gallic acid equivalent (mg/L) (Ertan et al., 2017).

#### 2.4.4 Antioxidant activity

The antioxidant activity was determined with the ABTS (2,2′‐azinobis‐3‐ethylbenzothiazoline‐6‐sulfonic acid) method (Barros et al., 2011; Silva, Ferreira, & Nunes, 2017). Before the analysis, 7.4 mM ABTS and 2.6 mM potassium persulfate solution were prepared and mixed at a 1:1 (v/v) ratio. After the mixture was stored at ambient temperature and in the dark for 12–16 h, the absorbance of the mixture was adjusted to 1.1 ± 0.02 by using methanol. The diluted solution was employed as the ABTS solution. A 150 ‐μL sample and 2850 μL ABTS solution were added to the centrifuge tube. The samples and standards were vortexed, and they were stored in the dark for 6 min. Measurements were made at 734 nm with a spectrophotometer (PG Instruments T80 UV/VIS Spectrophotometer, UK).

#### 2.4.5 ACE inhibitory activity

The ACE‐Inhibitory activity analysis of the samples was conducted with the ACE Kit‐WST A502 (Dojindo Molecular Technologies, Inc., Japan) based on the manufacturer's instructions (Şeker, 2017; Üstün‐Aytekin et al., 2020). Blank 1 (ultrapure water + enzyme + substrate), blank 2 (ultrapure water + substrate), and the samples (sample + enzyme + substrate) were prepared as described in the instructions. The Eppendorf tubes were stored at 37°C for 1 h, centrifuged at 1200 (g) for 10 min in the centrifuge, transferred to the microplates, and 200 μL indicator solution was added. Readings were conducted at 450 nm after the samples were kept for 10 min at ambient temperature. The ACE inhibitor activity was determined for each sample with the following equation (Şeker, 2017; Üstün‐Aytekin et al., 2020).

$\mathrm{ACE}\;\text{Inhibitor Activity}\;\left(\%\right)=\left[\left(A\;\text{Blank}1-A\;\text{Sample}\right)/\left(A\;\text{Blank}1-A\;\text{Blank2}\right)\right]\times 100.$

### 2.5 Physical analysis

The serum separation rate was measured with the method described by Temiz and Dağyıldız (2017). About 5 g of kefir were transferred to a centrifuge tube and centrifuged at 2500 g (Nüve NF 1200R, Turkey) for 10 min at 4°C. The resulting supernatant was weighed. The serum separation rate was determined by dividing the supernatant weight by the sample weight and multiplying the result by 100.

In color analysis, L*, a*, and b* were determined with the Hunter colorimeter (Hunter MiniScan Xe, Hunter Associates Laboratory Reston, USA). Based on the International Commission on Illumination (CIE) color system theory, the L* (brightness) varies between 0 and 100, a* reflects red/green (+a*/− a*), and b* indicates yellow/blue (+b*/−b*) (Koca, 2016).

### 2.6 Rheological analysis

Rheological measurements were conducted with the Brookfield Viscometer (Model DV‐II+ Viscometer, Brookfield Engineering Laboratories, Inc., USA) and SC4‐21 spindle. Viscosity analysis of elderberry kefir samples was conducted at 13 speeds (20, 40, 50, 60, 70, 80, 90, 100, 120, 140, 160, 180, and 200 rpm) at 4°C. Findings are presented as Pa.s. Thermal balance was achieved by adjusting the sample temperature to 4 ± 0.2°C with a cooling jacket. Based on the rheological measurements, increasing shear rate values formed the ascent curve, and decreasing shear rate values formed the descent curve. Based on the kefir sample output curve, consistency coefficients (*K*) and flow behavior index (*n*) were calculated with the Power Law Model presented below (Temen, 2018). Based on the power law,

σ=Kγn,

where *σ* is shear stress, *γ* is shear speed, *K* is the consistency coefficient, and *n* is the flow behavior index (Temen, 2018).

### 2.7 Microbiological analysis

The kefir sample (1 mL) was added to physiological saline under aseptic conditions. Decimal dilutions were prepared in sterile tubes that contained 9 mL saline. MRS agar (Biolife, Italy) was employed to determine the Lactobacillus count in elderberry kefir samples. Anaerocult A (Merck, Germany) was used for the anaerobic environment necessary for Lactobacillus microorganism growth, and the petri dishes were incubated at 37°C for 72 h. Lactobacillus count was determined by counting the colonies that developed at the end of the incubation period (Akbulut Ataman, 2020). M17 Agar (Biolife, Italy) was used to determine Lactococcus count, and petri dishes were incubated at 37°C for 48 h. The spill plate method was used to determine the microorganism count. The findings are presented as log cfu/mL (Kef & Arslan, 2021).

### 2.8 Sensory analysis

Sensory analysis of the kefir samples was conducted by 10 semi‐trained panelists (7 women and 3 men, aged 20–49). The panel included graduate students, undergraduate students, and Food Engineering Department faculty members. The panelists were regular consumers of fermented dairy products, including kefir, and experienced with the sensory properties of kefir. Samples were labeled with random three‐digit numbers and served in 30 mL plastic containers at 4°C. The panelists were asked to evaluate the appearance, consistency, taste/aroma of the samples, and state their general appreciation. Each parameter was scored with a 5‐point hedonic scale. During the analysis, water was served between each sample serving (Altuğ Onoğur & Elmacı, 2011; Çınar, 2019).

### 2.9 Statistical analysis

Statistical analyses were conducted with the analysis of variance. Statistical analyzes were conducted on the SPSS software (IBM Statistics Data Editor Version 20). An analysis of variance (ANOVA) was carried out to assess the impact of sample formulations and storage on the analyses. Factors determined to be significant were compared with the Duncan multiple comparison test (*p* < .05). The experiment was conducted in 2 replicates.

## 3 RESULTS AND DISCUSSION

### 3.1 Chemical composition

The protein, fat, and dry matter content and pH of the milk used in kefir production were 3.5%, 2.9%, 11.56%, and 6.6, respectively. The dry matter, water‐soluble dry matter and pH of the fresh elderberry fruit were 22.2%, 14.7, and 4.7. The dry matter and pH of the dried elderberry fruit were 92.6% and 4.50. The dry matter, fat, protein content, and pH of the kefir samples are presented in Table 2. It was determined that kefir samples with dried elderberry powder supplement included more dry matter when compared to the other kefir samples.

**TABLE 2:** The dry matter, fat, protein content, and pH of the kefir samples.

Sample	Dry matter (%)	Fat (%)	Protein (%)	pH
Control	11.09 ± 0.13^A^	2.65 ± 0.30	3.07 ± 0.31	4.37 ± 0.04
4‐FEPre	11.08 ± 0.32^A^	2.80 ± 0.21	3.23 ± 0.37	4.28 ± 0.06
2‐FEPre	11.07 ± 0.12^A^	2.67 ± 0.26	3.12 ± 0.19	4.29 ± 0.11
1‐DEPre	11.77 ± 0.16^C^	2.75 ± 0.26	3.44 ± 0.13	4.31 ± 0.09
0.5‐DEPre	11.36 ± 0.17^B^	2.67 ± 0.25	3.20 ± 0.36	4.35 ± 0.10
4‐FEPost	11.38 ± 0.20^B^	2.62 ± 0.22	3.06 ± 0.13	4.36 ± 0.06
2‐FEPost	10.96 ± 0.04^A^	2.72 ± 0.26	3.17 ± 0.20	4.37 ± 0.10
1‐DEPost	11.71 ± 0.04^C^	2.75 ± 0.23	3.22 ± 0.25	4.37 ± 0.02
0.5‐DEPost	11.36 ± 0.14^B^	2.82 ± 0.29	3.17 ± 0.45	4.25 ± 0.04

*Note*: The capital letters (A–C) indicate that the difference between samples was significant at the same storage period (*p* < .05).

There was no quantitative difference between the fat, protein content, and pH of the samples (*p* > .05). It was determined that the effect of the variations in the samples on dry matter content was statistically significant (*p* < .05). Among the kefir samples, 1‐DEPre sample had the highest dry matter content, while 2‐FEPostsample had the lowest dry matter content. It was determined that as the fruit supplementation ratio increased, the sample dry matter content increased. In a previous study, the dry matter content of the kefir samples with blueberry pulp was reported between 10.32% and 10.67% (Çınar, 2019).

The fat content findings of the present study were similar to those reported by Jadhav et al. (2023), who produced kefir with mango, jamun, and strawberry pulp supplements and protein findings were mostly higher. Jadhav et al. (2023) reported that the fat content of kefir samples was between 2.87 and 3.67%, and the protein content was between 2.79 and 3.36%. The protein content of the kefir with red fruit extract was quite similar to the protein content determined in our study (Erzhad et al., 2022).

### 3.2 Biological activity properties

Total phenolic content, total monomeric anthocyanin content, antioxidant activity, and ACE‐inhibitor activity of the kefir samples are presented in Table 3 based on time.

**TABLE 3:** Time‐dependent total phenolic substance, total monomeric anthocyanin content, antioxidant, and ACE‐inhibitor activity in kefir samples.

Sample	Storage	Total phenolic content (mg GAE/kg)	Total monomeric anthocyanin content (mg cyn‐3‐gly /kg)	Antioxidant activity (μmol TE/kg)	ACE‐inhibitor activity (%)
Control	1	167.94 ± 5.08^Aa^	—	16.69 ± 4.15^A^	92.78 ± 1.37^ABa^
	7	224.79 ± 5.54^Ab^	—	18.09 ± 3.64^A^	88.84 ± 0.38^Ba^
	14	204.54 ± 46.25^Aab^	—	18.35 ± 0.95^A^	90.46 ± 2.19^Aa^
4‐FEPre	1	440.81 ± 54.25^Ca^	78.14 ± 12.01^CDa^	43.32 ± 5.95^AB^	90.19 ± 0.24^Ab^
	7	407.21 ± 22.92^Ca^	68.71 ± 9.19^Da^	72.24 ± 6.49^B^	84.31 ± 0.20^Aa^
	14	501.15 ± 56.36^CDa^	85.32 ± 7.23^Ca^	66.91 ± 8.13^D^	89.17 ± 0.78^Ab^
2‐FEPre	1	301.81 ± 1.02^Ba^	37.61 ± 1.69^Aa^	36.38 ± 8.01^AB^	90.89 ± 0.68^Aa^
	7	335.05 ± 20.36^Ba^	28.38 ± 6.89^Ba^	47.55 ± 9.43^AB^	89.89 ± 0.12^Ca^
	14	352.36 ± 42.61^Ba^	35.60 ± 3.49^Aa^	39.87 ± 7.76^B^	91.28 ± 1.09^Aa^
1‐DEPre	1	448.89 ± 20.01^Ca^	66.12 ± 1.39^Cb^	65.10 ± 15.56^B^	94.74 ± 2.63^Bb^
	7	461.12 ± 43.96^Da^	49.63 ± 1.56^Ca^	72.44 ± 12.39^B^	84.07 ± 0.14^Aa^
	14	531.33 ± 57.22^Da^	53.84 ± 5.05^Ba^	61.25 ± 13.82^CD^	91.47 ± 0.06^Ab^
0.5‐DEPre	1	354.96 ± 35.68^Ba^	24.33 ± 5.34^Ab^	31.86 ± 5.98^AB^	94.62 ± 2.39^Bb^
	7	338.30 ± 11.13^Ba^	14.64 ± 2.36^Aa^	48.61 ± 10.14^AB^	84.23 ± 0.18^Aa^
	14	465.71 ± 51.19^BCDb^	22.28 ± 2.34^Aab^	39.85 ± 8.68^B^	90.84 ± 1.43^Ab^
4‐FEPost	1	430.06 ± 32.86^Ca^	88.20 ± 10.74^Db^	46.31 ± 3.82^AB^	92.42 ± 0.09^ABa^
	7	397.38 ± 39.09^Ca^	58.40 ± 2.91^CDa^	68.32 ± 9.95^B^	90.51 ± 0.28^CDa^
	14	458.62 ± 55.95^BCDa^	66.75 ± 9.07^Bab^	48.52 ± 8.52^BC^	90.60 ± 6.90^Aa^
2‐FEPost	1	302.93 ± 16.84^Ba^	43.78 ± 5.58^ABb^	29.66 ± 2.48^AB^	93.09 ± 2.15^ABa^
	7	299.54 ± 9.63^Ba^	26.21 ± 5.69^ABa^	47.22 ± 7.56^AB^	90.85 ± 0.55^Da^
	14	354.18 ± 69.96^Ba^	32.14 ± 7.45^Aab^	36.09 ± 9.74^B^	91.81 ± 0.88^Aa^
1‐DEPost	1	455.79 ± 6.43^Ca^	60.52 ± 6.39^BCab^	47.98 ± 11.58^AB^	92.35 ± 0.33^ABb^
	7	461.99 ± 38.64^Da^	63.99 ± 3.17^Db^	75.67 ± 17.81^B^	84.28 ± 0.15^Aa^
	14	505.34 ± 55.88^CDa^	53.93 ± 4.89^Ba^	56.13 ± 8.06^CD^	91.35 ± 1.88^Ab^
0.5‐DEPost	1	333.80 ± 6.86^Ba^	24.04 ± 1.47^Aa^	32.62 ± 13.67^AB^	92.42 ± 0.09^ABc^
	7	315.74 ± 11.13^Ba^	19.21 ± 4.87^ABa^	49.73 ± 15.50^AB^	83.80 ± 0.57^Aa^
	14	382.14 ± 43.01^BCa^	21.87 ± 5.15^Aa^	40.55 ± 9.09^B^	88.44 ± 0.72^Ab^

*Note*: The capital letters (A–D) indicate that the difference between samples was significant at the same storage period (*p* < .05). The lowercase letters (a,b) indicate that the difference (*p* < .05) between different storage periods was significant for the same sample.

Total phenolic content, total monomeric anthocyanin content, and antioxidant activity of the fresh elderberry fruit were 7930.5 mg GAE/kg, 3958.47 mg cyn‐3‐gly/kg, and 1176.5 μmol TE/kg. The total phenolic substance, total monomeric anthocyanin content, and antioxidant activity of the dried elderberry fruit were 35,004 mg GAE/kg, 16862.53 mg cyn‐3‐gly/kg, and 5034 μmol TE/kg, respectively.

It was determined that the impact of the formulation and storage on the phenolic content of kefir samples was statistically significant (*p* < .05). Total phenolic content increased during storage. Only the formulation affected the antioxidant activity (*p* < .05). It was determined that the antioxidant activity of the 1‐DEPost sample was the highest on the 7th day of storage. The lowest phenolic content was observed in the control sample on the 1st day of storage, and the highest phenolic content was observed in the kefir sample with 1% prefermentation dried elderberry powder supplement at the end of storage. The increase in fruit supplementation led to increases in total phenolic content and antioxidant activity.

Corona et al. (2016) produced kefir‐like fermented beverages with carrot, fennel, melon, onion, tomato, and strawberry juices and reported that the total phenolic content was between 101.83 mg GAE/L and 813.79 mg GAE/L in the samples, consistent with present study findings. Vicenssuto and de Castro (2020) reported higher antioxidant activities in kefir samples produced with mango peel when compared to the present study findings. They reported that the antioxidant activities in kefir with mango peel powder were between 136.09 μmol TE/g and 155.36 μmol TE/g and between 126.31 μmol TE/g and 142.07 μmol TE/g in kefir samples without mango peel powder.

The antioxidant activity and total phenolic content were generally higher when fruit was added before fermentation in kefir production. In a study on yogurt enriched with black mulberry, it was found that samples with fruit added before fermentation had a higher phenolic content and lower levels of antioxidant activity compared to yogurts with fruit added after fermentation. The researchers suggested that the increase in total phenolic content was likely due to the release of phenolics from fruit tissues or glycosides through the action of acids or microbial enzymes during fermentation. In this study, the low antioxidant activity values of samples with fruit added before fermentation were associated with the degradation of anthocyanins during incubation at 43°C and the interaction of phenolics with proteins or peptides produced during fermentation (Durmus et al., 2021). Some reports showed an increase in total phenolics and antioxidant activity during fermentation, which could be caused by the hydrolyzed complex phenolic compounds into simpler phenolic compounds and the formation of antioxidant metabolites during fermentation (Mhir et al., 2021; Sabokbar & Khodaiyan, 2016). In the present study, the addition of fruit before fermentation may have caused the formation of phenolic compounds and metabolites with antioxidant activity.

Kefir samples with dried elderberry powder supplement had higher total phenolic content and antioxidant activity when compared to other samples. In a study on elderberry wines, it was noted that the elderberry degrades during the fermentation process, leading to the release of more antioxidant substances (Cao et al., 2023). In this study, it was considered that kefir with dried elderberry powder produced more antioxidant substances and phenolic compounds from kefir with elderberry mash during the fermentation period.

The total phenolic content mostly increased during the storage period (except for some samples on the 7th day and the control kefir on the 14th day). The cause of this fluctuation may be due to the formation of new phenolic compounds by microbial enzymes, the degradation of some phenolic compounds, the decrease in free phenolics, and the balance between these factors (Durmus et al., 2021; Esatbeyoglu et al., 2023; Ramos et al., 2017). Antioxidant activity increased on the 7th day of storage and decreased on the 14th day of storage (except for the control sample). However, the impact of storage time on antioxidant activity was insignificant (*p* > .05). Taheur et al. (2023) found that phenolic content and antioxidant activity of kefir‐based dairy products added with red prickly pear powder increased during storage period.

Du et al. (2022) reported that the total phenolic content and anthocyanin content of yogurt supplemented with mulberry pomace increased during storage. The researchers noted that the antioxidant activity increased until the 21st day of storage but decreased on the 28th day. Intzirtzi et al. (2024) studied some properties of steam‐cooked beetroots during refrigerated storage. They found an increase in antioxidant activity with pigment content and a decrease in phenolic content during storage. Some studies have found a decrease in total phenolics and antioxidant activity during storage, which has been attributed to the complexation of phenolics with milk proteins (Cho et al., 2020; Durmus et al., 2021; Ramos et al., 2017).

During storage in kefir, fermentation metabolites such as peptides, free amino acids, fatty acids, enzymes, and other compounds have the ability to increase antioxidant capacity. However, the reduction in antioxidant potential may be due to the breakdown of phenolics by polyphenol oxidase under the influence of LAB and the interaction between proteins and polyphenols (Du et al., 2022). There are many parameters that affect protein‐phenolic interactions, such as temperature, pH, types of proteins, protein concentration, types and structures of phenolic compounds, salt concentration, and the addition of specific reactants. The complex formation of proteins and phenolics results from hydrogen bonding and hydrophobic interactions (Ozdal et al., 2013).

The effects of kefir formulation and storage time on total monomeric anthocyanin content and ACE inhibitor activity were statistically significant (*p* < .05). The highest monomeric anthocyanin content was determined in the kefir (4‐FEPost) sample with 4% postfermentation fresh elderberry supplement on the 1st day of storage; the lowest monomeric anthocyanin content was determined in the 0.5‐FEPost sample on the 7th day of storage. The total monomeric anthocyanin content was higher in the samples with fresh elderberry supplement when compared to the other kefir samples. It was determined that monomeric anthocyanin content increased with the rate of fruit supplementation. In a study on the freeze‐drying of elderberry anthocyanin extract, it was determined that anthocyanin recovery was 94%, and the physical structure and solubility were preserved (Brønnum‐Hansen & Flink, 1985). The higher anthocyanin content in products prepared from the mash may be due to the difference in anthocyanin content between fresh elderberry (3958.47 mg cyn‐3‐gly/kg) and its powdered form (16862.53 mg cyn‐3‐gly/kg), as well as the different addition rates.

Cordeiro et al. (2021) investigated the changes in anthocyanin content based on various cooking methods and the final product (jam, crumble, muffin, and mousse). They reported that the cooking method affected the anthocyanin content the loss was the highest in jam and the least in mousse (Cordeiro et al., 2021). In the present study, differences in anthocyanin content were expected between the kefirs produced with fresh fruit mash and dried elderberry powder.

Najgebauer‐Lejko et al. (2021) conducted a study with probiotic yogurt samples produced with various fruit puree supplements (10% elderberry, sea buckthorn and sloe puree) and reported that the total monomeric anthocyanin content was between 12.19 and 14.13 mg CGE (cyanidin‐3‐glucoside equivalent) /100 g in the probiotic yogurt samples with elderberry puree supplement during one‐month cold storage, between 4.77 and 9.96 mg CGE/100 g in samples with sloe puree, and between 2.15 and 2.34 mg CGE/100 g in samples with sea buckthorn puree.

Intzirtzi et al. (2024) reported that the stability of betalains in red beet was affected by a combination of internal (e.g., structural properties) and external factors (e.g., pH, temperature, organic acid content, etc.). It was also noted that storage under cold conditions preserved betalain stability and increased the amount of betacyanins during storage.

In another study, the effects of adding purified cyanidin or black currant extract before or after fermentation on drinkable yogurt were compared. It was found that drinkable yogurts with black currant polyphenols added before fermentation had higher anthocyanin content, while the addition of pure cyanidin before or after fermentation was not significant. The researchers indicated that there was no interaction between cyanidin and starter cultures during fermentation (Sun‐Waterhouse, Zhou, & Wadhwa, 2013).

Anthocyanins could easily degrade with environmental factors such as pH, temperature, enzymatic, and microbial activities (Raikos et al., 2018). In kefir production, fluctuations in total monomeric anthocyanin content were observed depending on the stage of fruit addition. The total sample monomeric anthocyanin content generally decreased on the 7th day of storage and increased on the 14th day of storage. In the present study, the fluctuations and the decrease in sample monomeric anthocyanin content could be associated with the changes in elderberry anthocyanin content due to the above‐mentioned factors.

ACE‐inhibitor activity was lower on the 7th day of storage and higher on the 14th day in all kefir samples; however, it never reached the level observed on the 1st day of storage. During storage, the highest ACE‐inhibitor activity was observed in the kefir sample (1‐DEPre) with 1% prefermentation dried elderberry supplement on the 1st day of storage, and the lowest ACE‐inhibitor activity was observed in the kefir sample with 0.5% postfermentation dried elderberry supplement (0.5‐DEPost) on the 7th day of storage. The use of different fruit forms in kefir had a slight effect on the change of ACE activity.

In a study conducted with goat milk kefir, it was reported that the ACE‐inhibitor activity was between 79.47% and 82.94% in the samples during the 14‐day storage (Shu et al., 2020). The authors argued that the changes in the ACE‐inhibitor activity in kefir samples were associated with the development of smaller molecules with the degradation of the ACE‐inhibitor peptide in kefir microorganisms during storage. In the present study, the ACE‐inhibitor activity of kefir samples was higher than those reported by Erkaya‐Kotan (2020), which ranged from 32.52 to 58.50 on yogurt incorporated with orange fiber.

Another study reported that ACE‐inhibitor activity was associated with proteolytic microorganism activities (Pihlanto et al., 2010). It could be suggested that sample‐dependent changes in ACE inhibitor activity during storage could be associated with microorganism activities.

Various factors, such as starter types, substrate composition, and fermentation processes, affect the formation of ACE‐I peptides during milk fermentation (Bakirci et al., 2023). While addition of elderberry mash after fermentation yielded a higher ACE inhibitor activity in the samples than addition before, the samples with elderberry powder added before fermentation exhibited more ACE inhibitor activity than that of the samples with fruit added after. The effect of fruit form on ACE inhibitor activity showed a fluctuating situation. There was very little difference between the samples.

In a study by Du et al. (2023) investigating the effect of mulberry pomace phenolic extract on yogurt, it was determined that mulberry pomace phenolic extract added after fermentation supported the release of active peptides and ACE‐I activity.

### 3.3 Physical properties

Time‐dependent serum separation, L*, b*, and a* values of kefir samples are presented in Table 4. Formulation and storage time significantly affected serum separation and a* values in kefir samples (*p* < .05). The lowest serum separation was determined in the 0.5‐DEPost sample on the 1st day of storage, and the highest serum separation was determined in the 2‐FEPost sample on the 7th day of storage.

**TABLE 4:** Time‐dependent serum separation, L*, b*, and a* values in kefir samples.

Sample	Storage	Serum separation (%)	L*	a*	b*
Control	1	52.85 ± 0.33^Aa^	88.37 ± 0.83^E^	−1.62 ± 0.01^Aa^	6.51 ± 0.10^B^
	7	53.26 ± 0.58^Ca^	87.07 ± 0.73^F^	−3.19 ± 0.21^Aa^	8.28 ± 0.18^G^
	14	53.34 ± 1.26^Aa^	86.84 ± 0.08^E^	−3.27 ± 0.11^Aa^	8.21 ± 0.13^F^
4‐FEPre	1	50.76 ± 1.47^Aa^	70.51 ± 1.36^BC^	7.37 ± 0.09^BCDa^	0.68 ± 0.12^A^
	7	52.48 ± 0.28^BCa^	72.01 ± 1.21^BCD^	4.95 ± 0.26^Ca^	1.82 ± 0.04^E^
	14	51.97 ± 1.63^Aa^	69.94 ± 1.35^B^	5.68 ± 0.05^DEa^	1.46 ± 0.08^D^
2‐FEPre	1	51.45 ± 1.80^Aa^	76.33 ± 0.38^D^	4.94 ± 0.03^Ba^	1.34 ± 0.16^A^
	7	53.19 ± 0.97^Ca^	76.96 ± 1.35^E^	2.78 ± 0.14^Ba^	2.64 ± 0.07^F^
	14	53.11 ± 0.19^Aa^	75.97 ± 0.84^D^	2.95 ± 0.23^Ba^	2.54 ± 0.19^E^
1‐DEPre	1	51.40 ± 0.89^Aa^	63.38 ± 0.59^A^	9.05 ± 0.08^CDb^	−0.96 ± 0.21^A^
	7	51.13 ± 1.55^ABa^	61.81 ± 0.37^A^	7.42 ± 0.15^Dab^	0.07 ± 0.14^B^
	14	51.57 ± 0.81^Aa^	65.17 ± 0.20^A^	6.87 ± 0.22^FGa^	0.00 ± 0.09^AB^
0.5‐DEPre	1	51.37 ± 1.03^Aa^	73.43 ± 0.67^CD^	5.80 ± 0.13^BCb^	−0.08 ± 0.03^A^
	7	51.21 ± 1.34^ABa^	72.46 ± 0.52^CD^	4.40 ± 0.23^Ca^	0.98 ± 0.09^D^
	14	51.70 ± 1.68^Aa^	72.10 ± 0.09^C^	4.25 ± 0.21^Ca^	0.94 ± 0.10^CD^
4‐FEPost	1	51.82 ± 2.25^Aa^	68.96 ± 0.35^B^	10.29 ± 0.10^Db^	−1.55 ± 0.16^A^
	7	53.43 ± 0.70^Ca^	69.26 ± 0.72^B^	7.29 ± 0.09^Da^	0.01 ± 0.06^B^
	14	52.62 ± 1.77^Aa^	70.17 ± 0.59^B^	6.42 ± 0.19^EFa^	0.49 ± 0.03^BC^
2‐FEPost	1	51.78 ± 1.15^Aa^	74.36 ± 1.06^CD^	7.02 ± 0.06^BCDb^	−0.05 ± 0.15^A^
	7	53.90 ± 0.68^Cb^	74.24 ± 1.84^DE^	5.33 ± 0.19^Cab^	0.82 ± 0.10^CD^
	14	52.61 ± 1.48^Aab^	74.29 ± 0.92^D^	4.45 ± 0.06^Ca^	1.35 ± 0.05^D^
1‐DEPost	1	50.69 ± 1.26^Aa^	64.69 ± 0.60^A^	9.85 ± 0.16^Db^	−1.89 ± 0.05^A^
	7	50.63 ± 2.00^Aa^	64.09 ± 0.06^A^	6.73 ± 0.15^Da^	−0.81 ± 0.11^A^
	14	52.60 ± 2.64^Aa^	64.84 ± 0.53^A^	7.41 ± 0.12^Gab^	−0.27 ± 0.08^A^
0.5‐DEPost	1	50.35 ± 2.16^Aa^	71.81 ± 0.90^BC^	7.03 ± 0.21^BCDa^	−0.49 ± 0.08^A^
	7	52.96 ± 0.53^Cb^	71.11 ± 1.15^BC^	5.31 ± 0.13^Ca^	0.42 ± 0.08^BC^
	14	53.52 ± 0.24^Ab^	70.12 ± 0.15^B^	5.38 ± 0.03^Da^	0.61 ± 0.03^BC^

*Note*: The capital letters (A–G) indicate that the difference between samples was significant at the same storage period (*p* < .05). The lowercase letters (a,b) indicate that the difference (*p* < .05) between different storage periods was significant for the same sample.

In a study conducted with kefirs produced with arbutus and tamarind, it was determined that syneresis varied between 25.07% and 45.30% during storage (Kulaksız Günaydı & Ayar, 2022).

Although storage and sample differences were statistically significant on serum separation, serum separation values were quite close to each other. Syneresis of kefir increased during storage period. A decrease in pH can also enhance protein–protein and protein–phenolic interactions, leading to an increase in serum separation during storage (Durmus et al., 2021).

The impact of sample formulation on L* and b* was statistically significant (*p* < .05). The control sample exhibited the highest L* and b*, while the 1‐DEPost sample exhibited the lowest L* and b* at the end of storage. L* and b* decreased with the increase in fruit supplementation. Dried fruit powder supplementation led to a lower L* when compared to fresh fruit supplementation. As seen in the table, certain samples had a positive b* (yellow), while others had a negative b* (blue). The samples had a positive b* (except for the 1‐DEPost sample) on the 7th and 14th days of storage.

Sample formulation and storage period had statistically significant effects on a* (*p* < .05). Kefir samples with elderberry supplement exhibited a positive a*. It was determined that the 4‐FEPost sample exhibited the highest positive a* at the beginning of storage.

In the study, the lowest positive a* was observed in the 2‐FEPre sample on the 7th day of storage.

Dimitreli et al. (2019) investigated the effect of various rates of postfermentation fir honey and pomegranate juice supplementation to kefir with various fat content on the color and reported L* values between 71.4 and 86.0, a* values between −2.05 and 3.15, and b* values between 0.20 and 7.45. The authors reported that the increase in pomegranate juice supplementation decreased the L* and increased the a*.

a* values of kefir samples decreased during storage period (except some samples on the 14th day). A similar decrease was reported by Silva, Silva, et al. (2017) for fermented dairy beverages with blueberry juice. Researchers explained that the decrease in a* values could be attributed to the saturation of pigments, which deplete the substrate pool necessary for complexation with anthocyanins, leading to a destabilizing effect on color.

Ścibisz et al. (2019) conducted a study with fruit yogurt that they supplemented with various fruits (strawberries, sour cherries and blueberries), and reported that the redness decreased during storage and the product color changed from red to orange. This change was probably due to yellow and brown polymerization due to the degradation and/or development of anthocyanins. They reported that several factors such as pH, enzyme, ascorbic acid content, and the time of fruit supplementation could have been effective on the product color.

The supplementation of elderberry in different forms, amounts, and times in kefir production could affect the color of the kefir samples based on anthocyanin content. The significant effect of storage on a* could be due to changes in anthocyanins and the amount of anthocyanins during storage.

L* and b* were mostly higher in kefir with prefermentation fruit supplement when compared to postfermentation fruit supplementation. L* and b* values of the samples fluctuated during storage, and the effect of storage time on color values was not significant. The samples with elderberry added after fermentation had higher a* values.

In another study, it was reported that adding fruit before fermentation caused an increase in L* and b* values and a decrease in a* values in tamarillo‐added yogurts. The researchers indicated that the fermentation process might have affected the color results due to the absorption of water by dietary fibre and polyphenols in tamarillo and that the decrease in acidity could have led to the deterioration of the color of natural pigments, such as anthocyanins, in the yogurt matrix (Diep et al., 2022). In this present study, the increase in acidity may have caused changes in the anthocyanins in elderberry and alterations due to the absorption of water by the phenolic compounds in elderberry.

### 3.4 Rheological properties

The rheological consistency coefficient (*K*) and flow behavior index (*n*) measurements conducted on the 1st, 7th, and 14th days of storage at 4°C are presented in Table 5. It was determined that the flow behavior index of the samples was less than 1 (*n* < 1) based on the Power Law and they exhibited pseudoplastic flow. *R* ^2^ values of the samples were between 0.98 and 0.99.

**TABLE 5:** Time‐dependent sample consistency coefficients (*K*) and flow behavior indices (*n*).

	Storage (day)					
	1		7		14	
Kefir Sample	Flow behavior index (*n*)	Consistency coefficients (*K*) Pa.s	Flow behavior index (*n*)	Consistency coefficients (*K*) Pa.s	Flow behavior index (*n*)	Consistency coefficients (*K*) Pa.s
Control	0.26 ± 0.08^a^	5.92 ± 0.99^a^	0.28 ± 0.02^a^	5.32 ± 1.10^a^	0.24 ± 0.04^a^	7.39 ± 1.68^a^
4‐FEPre	0.46 ± 0.12^a^	1.38 ± 0.41^a^	0.28 ± 0.02^a^	5.46 ± 0.12^b^	0.23 ± 0.02^a^	7.53 ± 1.61^b^
2‐FEPre	0.46 ± 0.03^b^	1.44 ± 0.53^a^	0.32 ± 0.07^ab^	4.45 ± 1.25^ab^	0.23 ± 0.02^a^	7.44 ± 1.32^b^
1‐DEPre	0.35 ± 0.25^a^	4.03 ± 1.41^a^	0.23 ± 0.01^a^	4.83 ± 0.02^a^	0.21 ± 0.00^a^	5.60 ± 0.18^a^
0.5‐DEPre	0.36 ± 0.06^a^	1.95 ± 0.87^a^	0.25 ± 0.05^a^	4.68 ± 1.59^ab^	0.23 ± 0.01^a^	7.06 ± 0.82^b^
4‐FEPost	0.34 ± 0.04^a^	2.71 ± 0.72^a^	0.27 ± 0.02^a^	5.33 ± 0.94^b^	0.25 ± 0.01^a^	6.58 ± 0.50^b^
2‐FEPost	0.44 ± 0.07^a^	1.75 ± 0.74^a^	0.32 ± 0.12^a^	4.80 ± 1.13^a^	0.23 ± 0.02^a^	7.98 ± 1.59^a^
1‐DEPost	0.33 ± 0.14^a^	2.96 ± 0.58^a^	0.24 ± 0.04^a^	5.16 ± 1.32^a^	0.18 ± 0.00^a^	7.51 ± 0.15^a^
0.5‐DEPost	0.40 ± 0.12^a^	2.29 ± 0.48^a^	0.23 ± 0.02^a^	6.27 ± 1.45^ab^	0.16 ± 0.00^a^	8.55 ± 0.74^b^

Abbreviations: The lowercase letters (a,b) indicate that the difference (*p* < .05) between different storage periods was significant for the same sample.

It was determined that the impact of kefir formulation on the flow behavior index and consistency coefficient was statistically insignificant (*p* > .05). However, the impact of storage on the consistency coefficient and flow behavior index was statistically significant (*p* < .05).

During storage, the sample flow behavior index (*n*) generally decreased, and the consistency coefficient increased. The flow behavior index showed a large decline on the 7th day and a small decline on the 14th day. The control sample exhibited the highest consistency coefficient on the 1st day of storage, while the kefir sample produced with 0.5% postfermentation dry elderberry powder supplement exhibited the highest consistency coefficient on the 14th day of storage.

Vimercati et al. (2020) reported that based on the rheological measurements conducted on the coffee‐flavored kefir at 18°C, the consistency coefficient (*K*) varied between 1.556 and 5.009 Pa.s, the flow behavior index varied between 0.034 and 0.355, and the samples exhibited pseudoplastic flow behavior.

A study conducted on kefir determined that pomegranate juice and pomegranate peel extract supplements decreased viscosity, increased the flow behavior index, and improved rheological properties in kefir samples (Lagouri et al., 2019).

In the study where the impact of flower and pine honey supplements on the rheological properties of kefir was investigated, it was reported that the flow behavior index (*n*) and consistency index (*K*) varied between 0.3711 and 0.4991, 130 and 204 mPa.s, respectively. Honey kefir samples exhibited non‐Newtonian behavior (Doğan, 2011).

Lubbers et al. (2004) reported that the viscosity and consistency coefficient of the strawberry‐flavored fat‐free yogurts increased, and the flow behavior index decreased during storage.

In a study where the effect of pre‐ and postfermentation strawberry juice supplementation was investigated on the rheological properties of fermented goat milk, the samples exhibited pseudoplastic behavior, postfermentation fruit juice supplement had no obvious effect on the rheological properties, and prefermentation fruit juice supplement significantly affected viscosity. Researchers evaluated that the interactions between pectin in fruit juice and exopolysaccharides released by starter cultures affected the structure during fermentation (Wang et al., 2019).

Durmus et al. (2021) stated that the addition of fruit to yogurt after fermentation resulted in a higher consistency coefficient (*k*) and a lower flow behavior index (*n*) compared to yogurt with fruit added before fermentation. They suggested that adding fruit to milk likely hindered the development of protein–protein interactions and locally disrupted the gel network, thereby weakening the overall structure of the resulting gel.

In the present study, the effect of the fruit addition stage on rheological properties is consistent with the above study.

In a study on passion fruit yogurts, it was reported that the addition of passion fruit increased the gel strength due to the interaction of phenolic compounds, negatively charged pectin, or plant polysaccharides in the fruit juice with casein. Additionally, they found that when the concentration of passion fruit juice exceeded 7.5%, the gel strength weakened, which was attributed to the high organic content affecting the casein gel (Ning et al., 2021). In the present study, the timing of fruit addition and the form of the fruit did not have a significant impact on the rheological properties of the product.

Similarly, a study conducted on tamarillo‐supplemented yogurt indicated that adding fruit before or after fermentation did not affect the consistency coefficient index and flow behavior index, but these parameters changed as the addition rate varied (Diep et al., 2022).

### 3.5 Microbiological properties

The time‐dependent Lactobacillus and Lactococcus counts in kefir samples are presented in Table 6. The effect of sample differences and storage time on the lactobacilli count was statistically significant in kefir samples (*p* < .05). Lactobacillus count decreased during storage in the samples, and the sample with 4% prefermentation fresh elderberry supplement had the highest Lactobacillus count on the 14th day of storage. It was determined that kefir samples with elderberry fruit mostly had higher Lactobacillus counts when compared to the control sample.

**TABLE 6:** Time‐dependent Lactobacillus and Lactococcus count in kefir samples.

Sample	Storage	Lactobacillus (log cfu/mL)	Lactococcus (log cfu/mL)
Control	1	9.03 ± 0.07^BCb^	9.09 ± 0.11^a^
	7	8.32 ± 0.28^ABa^	8.58 ± 0.28^a^
	14	8.19 ± 0.36^Aa^	8.57 ± 0.44^a^
4‐FEPre	1	9.16 ± 0.11^Cb^	9.15 ± 0.06^b^
	7	8.79 ± 0.13^Da^	8.88 ± 0.20^ab^
	14	8.61 ± 0.20^Aa^	8.82 ± 0.26^a^
2‐FEPre	1	9.06 ± 0.05^BCb^	8.99 ± 0.09^a^
	7	8.81 ± 0.22^Db^	9.06 ± 0.15^a^
	14	8.36 ± 0.34^Aa^	8.51 ± 0.57^a^
1‐DEPre	1	9.13 ± 0.03^Cb^	9.17 ± 0.07^b^
	7	8.59 ± 0.09^BCDa^	8.70 ± 0.22^ab^
	14	8.32 ± 0.33^Aa^	8.59 ± 0.53^a^
0.5‐DEPre	1	8.89 ± 0.08^Ab^	9.05 ± 0.03^a^
	7	8.51 ± 0.14^ABCDa^	8.82 ± 0.17^a^
	14	8.22 ± 0.33^Aa^	8.51 ± 0.62^a^
4‐FEPost	1	9.16 ± 0.02^Cc^	9.12 ± 0.06^b^
	7	8.67 ± 0.04^CDb^	8.74 ± 0.11^ab^
	14	8.23 ± 0.31^Aa^	8.63 ± 0.43^a^
2‐FEPost	1	8.94 ± 0.17^ABc^	9.02 ± 0.19^a^
	7	8.39 ± 0.19^ABCb^	8.50 ± 0.40^a^
	14	7.93 ± 0.20^Aa^	8.52 ± 0.55^a^
1‐DEPost	1	9.10 ± 0.02^Cb^	9.10 ± 0.03^a^
	7	8.67 ± 0.18^CDab^	8.63 ± 0.44^a^
	14	8.17 ± 0.90^Aa^	8.53 ± 0.63^a^
0.5‐DEPost	1	9.15 ± 0.08^Cb^	9.24 ± 0.03^b^
	7	8.24 ± 0.27^Aa^	8.67 ± 0.34^ab^
	14	8.13 ± 0.31^Aa^	8.32 ± 0.68^a^

*Note*: The capital letters (A–D) indicate that the difference between samples was significant at the same storage period (*p* < .05). The lowercase letters (a–c) indicate that the difference (*p* < .05) between different storage periods was significant for the same sample.

Elderberry has rich flavonols and phenolic acid content, including quercetin, kaempferol, isorhamnetin‐glycosides, and quercetin‐3‐O‐rutinoside. Although polyphenols are known to negatively affect bacterial viability, they have been reported to support the growth of certain LAB. A study on elderberry drinks investigated the effects of certain Lactobacillus strains on polyphenol metabolism. It was reported that *Lb. rhamnosus* and *Lb. plantarum* strains were quite effective on polyphenol metabolism, and under the influence of these microorganisms, certain polyphenols decreased (hydroxycinnamic acid and hydroxybenzoic acid), while others increased (dihydroxy‐caffeic acid, catechol, and anthocyanin) (Ricci et al., 2019).

It was reported that the decrease in the LAB count during storage was associated with the decrease in developmental nutrients required by the microorganisms (Ozcelik et al., 2021).

The impact of storage duration on the Lactococcus count in kefir was statistically significant (*p* < .05), the impact of sample formulation on the Lactococcus count was statistically insignificant (*p* > .05). It was determined that the Lactococcus count decreased in most samples during storage. At the end of storage, the highest Lactococcus count was identified in the kefir sample (4‐FEPre) with 4% prefermentation elderberry fruit supplement.

Say et al. (2019) reported that the lactobacilli count decreased during the 21‐day storage period in kefir samples produced with strawberry and apricot flavor supplements, and the Lactobacillus count varied between 6.04 and 6.61 log cfu/mL in strawberry‐flavored kefir samples, and between 6.85 and 6.20 log cfu/mL in apricot‐flavored kefir samples.

Özcan et al. (2018) reported lower Lactococcus and Lactobacillus counts in plain and fruit‐flavored samples when compared to our microbiological findings. The authors determined that the Lactococcus and Lactobacillus counts decreased with storage. It was determined that the decrease in microorganism count during the storage of plain kefir was higher when compared to the fruit‐flavored kefir. Fruit glucose, fructose, dietary fiber, vitamin, and phenolic content positively affect microorganism viability in fruit‐flavored kefir.

Harmankaya et al. (2019) produced kefir with 20% fruit (strawberries, apricots, bananas) supplementation during production. Lactococcus spp. content was 6.68–9.08 log cfu/mL in the control samples, 7.56–9.58 log cfu/mL in the strawberry kefir samples, 7.30–8.81 log cfu/mL in the banana kefir samples, and 8.70–9.30 log cfu/mL in the apricot kefir samples.

The samples with fruit added before fermentation were found to have higher Lactobacillus counts compared to the samples with fruit added after fermentation. Polyphenols have varying levels of antibacterial or antimicrobial effects, and the addition of polyphenols before fermentation can have positive or negative impacts on bacterial growth. Factors such as the chemical structure of the added bioactive polyphenols, their antioxidant activity, and the characteristics of the bacteria influence bacterial growth (Sun‐Waterhouse, Zhou, & Wadhwa, 2013).

Aydin et al. (2022) indicated that anthocyanin has a complex effect on the kefir microbiome, with the potential to have dose‐related growth‐promoting effects on several bacteria in kefir (e.g., *Lb kefiri, L. mesenteroides*) and dose‐related inhibitory effects on others (e.g., *L. lactis*).

Another study found that adding fruit extract or pure phenolic compounds before fermentation resulted in higher total phenolic content and better support for the development of bacterial cultures compared to adding them after fermentation. They attributed these findings to the formation of smaller phenolic compounds during the fermentation process (Sun‐Waterhouse et al., 2012; Sun‐Waterhouse, Zhou, & Wadhwa, 2013).

### 3.6 Sensory properties

Sensory analysis of the kefir samples is presented in Table 7. Sensory analysis scores demonstrated that the effect of sample formulation on appearance and consistency was statistically significant (*p* < .05). Furthermore, storage time did not have a statistically significant impact on sample appearance and consistency (*p* > .05). Sample formulation and storage time had an impact on taste–aroma and general appreciation.

**TABLE 7:** Sample sensory analysis scores.

Sample	Storage	Appearance	Consistency	Taste–aroma	General appreciation
Control	1	4.30 ± 0.73^C^	4.60 ± 0.59^B^	4.45 ± 0.68^Ba^	4.60 ± 0.59^Ba^
	7	4.40 ± 0.75^C^	4.40 ± 0.68^B^	4.60 ± 0.75^Ca^	4.50 ± 0.88^Ba^
	14	4.40 ± 0.68^D^	4.30 ± 0.65^B^	4.30 ± 0.80^Ba^	4.30 ± 0.80^Ba^
4‐FEPre	1	4.35 ± 0.74^C^	3.80 ± 0.76^A^	3.45 ± 1.05^Aa^	3.45 ± 1.05^Aa^
	7	3.90 ± 0.64^ABC^	3.95 ± 0.94^AB^	3.35 ± 0.87^Aa^	3.50 ± 0.88^Aa^
	14	4.10 ± 0.68^CD^	4.25 ± 0.71^B^	3.35 ± 0.74^Aa^	3.40 ± 1.23^Aa^
2‐FEPre	1	3.55 ± 0.88^A^	3.65 ± 0.81^A^	3.30 ± 1.03^Aa^	3.25 ± 0.91^Aa^
	7	3.70 ± 0.86^A^	3.75 ± 1.11^A^	3.55 ± 1.05^ABa^	3.50 ± 1.14^Aa^
	14	3.20 ± 1.0^A^	3.55 ± 1.05^A^	3.10 ± 1.16^Aa^	3.10 ± 1.11^Aa^
1‐DEPre	1	4.40 ± 0.99^C^	4.05 ± 0.99^A^	3.50 ± 1.31^Aa^	3.70 ± 1.41^Aa^
	7	4.25 ± 0.91^ABC^	4.00 ± 0.85^AB^	3.80 ± 0.83^ABa^	3.95 ± 1.05^ABa^
	14	4.35 ± 0.58^D^	3.90 ± 0.78^AB^	3.60 ± 1.04^Aa^	3.75 ± 0.91^ABa^
0.5‐DEPre	1	3.75 ± 0.91^AB^	3.90 ± 0.78^A^	3.45 ± 0.68^Aa^	3.60 ± 0.68^Aa^
	7	3.80 ± 0.89^AB^	4.05 ± 0.88^AB^	4.15 ± 0.98^BCb^	3.85 ± 0.98^ABa^
	14	3.75 ± 1.01^BC^	3.65 ± 0.93^A^	3.55 ± 0.94^Aa^	3.45 ± 0.99^Aa^
4‐FEPost	1	4.35 ± 0.58^C^	4.00 ± 0.79^A^	3.70 ± 1.03^Aa^	3.70 ± 0.80^Aab^
	7	3.85 ± 0.67^ABC^	4.20 ± 0.83^AB^	3.90 ± 0.71^ABa^	4.05 ± 0.68^ABb^
	14	4.20 ± 0.41^CD^	4.00 ± 0.72^AB^	3.60 ± 0.88^Aa^	3.40 ± 0.99^Aa^
2‐FEPost	1	4.10 ± 0.64^BC^	4.00 ± 0.97^A^	3.30 ± 1.17^Aa^	3.65 ± 1.03^Aa^
	7	3.95 ± 0.60^ABC^	4.15 ± 0.81^AB^	3.50 ± 1.10^Aa^	3.80 ± 1.00^Aa^
	14	3.50 ± 0.68^AB^	3.55 ± 0.88^A^	2.95 ± 1.23^Aa^	3.35 ± 0.98^Aa^
1‐DEPost	1	4.40 ± 0.75^C^	4.05 ± 0.68^A^	3.60 ± 0.99^Aa^	3.70 ± 1.08^Aa^
	7	4.30 ± 0.73^BC^	4.30 ± 0.86^AB^	3.90 ± 0.91^ABab^	4.00 ± 0.91^ABab^
	14	4.50 ± 0.51^D^	4.10 ± 0.71^AB^	4.35 ± 0.74^Bb^	4.35 ± 0.67^Bb^
0.5‐DEPost	1	3.75 ± 0.85^AB^	3.55 ± 0.88^A^	3.55 ± 1.05^Aa^	3.45 ± 0.99^Aa^
	7	3.90 ± 0.78^ABC^	3.70 ± 0.73^A^	3.80 ± 0.89^ABa^	3.85 ± 0.81^ABa^
	14	4.00 ± 0.91^BCD^	4.25 ± 0.78^B^	3.40 ± 0.68^Aa^	3.50 ± 0.68^Aa^

*Note*: The capital letters (A–D) indicate that the difference between samples was significant at the same storage period (*p* < .05). The lowercase letters (a,b) indicate that the difference (*p* < .05) between different storage periods was significant for the same sample.

In the beginning of storage, the control sample received the highest consistency, general appreciation, and taste–aroma scores. At the end of storage, the sample with 1% postfermentation dried elderberry supplement received the highest appearance, taste–aroma, and general appreciation scores. It was determined that the sample with 2% prefermentation fresh elderberry supplement received lower sensory scores. The general appreciation scores varied between 3.10 and 4.60, and the samples with dried elderberry powder supplement were appreciated more than the samples with fresh elderberry.

In a study conducted with kefir with encapsulated blackberry juice, the most preferred sample was the sample with 7.5% blackberry juice supplement, and the least appreciated sample was the sample with 1% blackberry juice supplement. It was determined that the increase in blackberry juice supplement improved appreciation (Travičić et al., 2023). In the present study, it was observed that the increase in elderberry supplementation improved appreciation.

In a study conducted with a kefir drink with two types of passion fruit, it was reported that appreciation was affected since the panelists were not familiar with the fruit; however, acceptability was still reasonable (Mendes et al., 2021).

The general appreciation scores of the samples (except the control sample) increased on the 7th day of storage, while they decreased on the 14th day. This finding was consistent with previous reports. Yılmaz et al. (2006) conducted a study with kefir products with various fruit flavors (blackberry, raspberry, and strawberry), and best acceptability was reported on the 4th day, and acceptability decreased significantly on the last day (day 10) of storage.

## 4 CONCLUSION

It was reported that the beneficial effects of black elderberry fruit on human health depend on the presence of bioactive substances such as anthocyanins, flavonoids, polyphenols, and phenolic acids. Due to its unique natural color and rich nutritional content, elderberry fruit has been used in the production of various food products. In kefir production, samples with fruit added before fermentation had higher phenolic content, antioxidant activity, L*, and b* values compared to those with fruit added after fermentation. It was determined that the kefir sample with the highest ACE‐inhibitor activity was 1‐DEPre (kefir with 1% prefermentation dried elderberry fruit supplement). Total monomeric anthocyanin content increased with the increase in elderberry supplementation, and the fresh fruit supplement was more effective on total monomeric anthocyanin content when compared to the dried elderberry supplement. Lactobacillus and Lactococcus counts in kefir samples produced with various pretreated elderberries were similar and decreased during storage. Elderberry fruit supplementation rate affected the appearance score, and the increase in the rate improved the appearance scores. Kefir samples with dried fruit supplement had higher antioxidant activity, total phenolic content, dry matter content, and general taste scores. In terms of biological activity, microbiological aspects, and color values (L* and b* values), it is observed that the addition of elderberry before fermentation better results. It could be suggested that dried elderberries should be preferred in kefir production due to ease of storage. Additionally, the addition of elderberry before fermentation is also among our recommendations. In the present study, the employment of black elderberry fruit in kefir production extended the consumption range of the black elderberry fruit and improved the functional properties of kefir drink.

## AUTHOR CONTRIBUTIONS

**Ümran Barazi:** Formal analysis (equal); investigation (equal); methodology (equal); writing – original draft (supporting). **Seher Arslan:** Data curation (equal); investigation (equal); methodology (equal); supervision (lead); writing – original draft (lead); writing – review and editing (lead).

## CONFLICT OF INTEREST STATEMENT

The authors declare that they have no competing interests.

## Data Availability

The data that support the findings of this study are available from the corresponding author upon reasonable request.
